# Retraction Note: Role of short-wavelength blue light in the formation of cataracts and the expression of caspase-1, caspase-11, Gasdermin D in rat lens epithelial cells: insights into a novel pathogenic mechanism of cataracts

**DOI:** 10.1186/s12886-022-02416-9

**Published:** 2022-04-26

**Authors:** Yamin Wang, Min Zhang, Ying Sun, Xiaohui Wang, Zhaowei Song, Huazhang Li, Kexin Liu, Zhijian Li

**Affiliations:** 1 Department of Ophthalmology, The First Affiliated Hospital, Harbin Medical University, 143 Yiman Street, Nangang District, Harbin, China; 2 Department of Ophthalmology, The 2nd Hospital of Heilongjiang, Harbin, China

**Retraction Note: BMC Ophthalmol 20, 289 (2020)**

**https://doi.org/10.1186/s12886-020-01565-z**

The Editor has retracted this article. After publication, concerns were raised regarding the presented data. Specifically, the flow cytometry plots in Fig. 3a appear not to be representative of the experimental results based on the full data provided by the authors. In addition, the authors used an unsuitable method (Annexin V/PI flow cytometry assay) to distinguish pyroptosis from apoptosis. Furthermore, the western blotting images presented in Fig. 7 do not appear to match the quantitative data in the bar graph. The Editor therefore no longer has confidence in the presented data and the conclusions of this article.

Zhijian Li agrees to this retraction. Yamin Wang has agreed to this retraction but not to the wording of this retraction notice. Min Zhang, Ying Sun, Xiaohui Wang, Zhaowei Song, Huazhang Li and Kexin Liu have not responded to any correspondence from the editor or publisher about this retraction.

